# Evidence for Mesenchymal−Epithelial Transition Associated with Mouse Hepatic Stem Cell Differentiation

**DOI:** 10.1371/journal.pone.0017092

**Published:** 2011-02-11

**Authors:** Bin Li, Yun-Wen Zheng, Yuuki Sano, Hideki Taniguchi

**Affiliations:** Department of Regenerative Medicine, Yokohama City University Graduate School of Medicine, Yokohama, Japan; Instituto Nacional de Câncer, Brazil

## Abstract

Mesenchymal−epithelial transition events are related to embryonic development, tissue construction, and wound healing. Stem cells are involved in all of these processes, at least in part. However, the direct evidence of mesenchymal−epithelial transition associated with stem cells is unclear. To determine whether mesenchymal−epithelial transition occurs in liver development and/or the differentiation process of hepatic stem cells *in vitro*, we analyzed a variety of murine liver tissues from embryonic day 11.5 to adults and the colonies derived from hepatic stem/progenitor cells isolated with flow cytometry. The results of gene expression, immunohistochemistry and Western blot showed that as liver develops, the expression of epithelial markers such as Cytokeratin18 and E-cadherin increase, while expression of mesenchymal markers such as vimentin and N-cadherin decreased. On the other hand, in freshly isolated hepatic stem cells, the majority of cells (65.0%) co-express epithelial and mesenchymal markers; this proportion is significantly higher than observed in hematopoietic cells, non-hematopoietic cells and non-stem cell fractions. Likewise, in stem cell-derived colonies cultured over time, upregulation of epithelial genes (Cytokeratin-18 and E-cadherin) occurred simultaneously with downregulation of mesenchymal genes (vimentin and Snail1). Furthermore, in the fetal liver, vimentin-positive cells in the non-hematopoietic fraction had distinct proliferative activity and expressed early the hepatic lineage marker alpha-fetoprotein.

**Conclusion:**

Hepatic stem cells co-express mesenchymal and epithelial markers; the mesenchymal−epithelial transition occurred in both liver development and differentiation of hepatic stem/progenitor cells *in vitro*. Besides as a mesenchymal marker, vimentin is a novel indicator for cell proliferative activity and undifferentiated status in liver cells.

## Introduction

Mesenchymal-epithelial transition (MET) events are defined as those in which mesenchymal cells lose their motile, migratory properties and acquire cell polarity and adhesion to epithelia. MET and the reverse process, epithelial mesenchymal transition (EMT), both occur in normal tissue, including gastrulating and regenerating tissue, as well as abnormal tissues of fibrotic organs or tumors [1], [2]. Thus, it is necessary to reveal the relationship between EMT/MET and stem cells. Indeed, EMT drives mammary epithelial cells to de-differentiate into mammary stem cells and cancer stem cells which are mesenchymal-like [3]. Moreover, induced pluripotent stem cells (iPSCs) are derived from mouse embryonic fibroblasts (MEF) by MET at the early stage of reprogramming [4]–[6]. These results suggest the possibility that MET is associated with stem cell activities.

Recent work reported that rat hepatic oval cells (hepatic progenitors) express mesenchymal markers; this result indicated but not fully confirmed that MET existed in the process of hepatic progenitor cell differentiation [7]. On the other hand, hepatocytes, cholangiocytes and liver are known to undergo EMT under sophisticated regulation [8]–[12]. Furthermore, according to the pathological progression from normal liver to hepatic cirrhosis and then to hepatic carcinoma, EMT may be associated [8], [13]–[15]. Therefore, MET in hepatic stem cells is important to multiple processes, including liver development, regeneration, and chronic liver injury.

In order to identify EMT/MET, vimentin is widely applied as a mesenchymal indicator [1], [3], [16]–[19]. Vimentin is an intermediate filament protein functionally involved in maintaining the structure of mesenchymal cells [20]. In addition to serving as a marker in EMT/MET, vimentin plays a versatile role in cancer cell motility. In prostate cancer cells, for example, vimentin links the motility and un-differentiated state of cells [21]. Notwithstanding, in breast cancer, vimentin mRNA expression is related to mesenchymal cell shape and motility [22]. In normal tissue injury, depletion of vimentin delays wound healing by mesenchymal fibroblasts trans-differentiating into epithelia [23]. Little is known about the relationship between vimentin expression and normal cell activation.

To address these questions, we investigated the mesenchymal characteristics of fetal liver cells and observed MET in developing mouse liver. Furthermore, based on our previous work [24], we isolated hepatic stem cells with flow cytometry in order to study MET in stem cell differentiation. Importantly, we revealed that vimentin is associated with proliferative activity in liver cells.

## Results

### Mesenchymal−epithelial transition is a spontaneous process involved in mouse liver development

**Figure 1:**
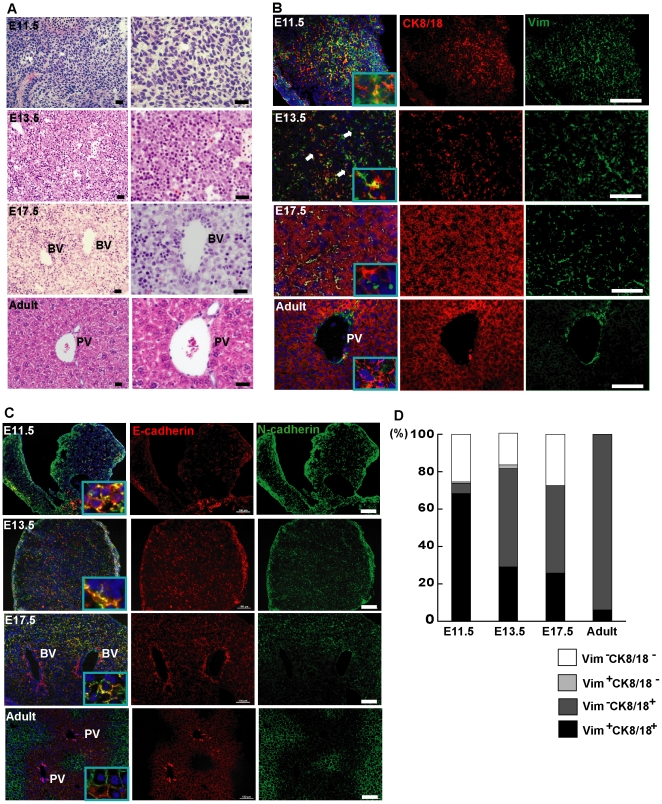
Mesenchymal−epithelial transition occurs in developing mouse liver. (A) Phenotype changes of liver cells according to development stages. Representative images showed hematoxylin/eosin staining of livers from C57BL/6J mice at E11.5, 13.5 and 17.5 and 8 weeks (Adult) after birth. Right panels showed the magnified pictures. (B) Immunofluorescence for simultaneous detection of CK8/18 (epithelial) and vimentin (mesenchymal); (C) E-cadherin (epithelial) and N-cadherin (mesenchymal) in livers from mice in (A). Arrows in (B) showed the CK8/18 and vimentin overlapping cells. (D) The ratio for CK8/18 and/or vimentin expressing non-hematopoietic liver cells from mice in indicated development stages. Quantitative analyses were based on immunofluorescence staining. These images showed gain of epithelial characters and loss of mesenchymal characters within mouse liver development. Vim: vimentin. E: embryonic day. BV: Blood vessel. PV: Portal vein. Scale bars = 100 µm.

To investigate mesenchymal-epithelial transition in liver development, we analyzed the phenotype of cells and MET molecules in developing and adult mouse livers (Figure 1). ED11.5 liver cells are mesenchymal-like with lacking cell−cell connections (Figure 1A). As development proceeds, from ED11.5 to adult mouse, liver cells exhibited the change of cell shape from spindle to polygon, with larger size and tighter intercellular connections (Figure 1A). Moreover, an expansion of epithelial markers CK8/18 (Figure 1B and 1D; Table 1) and ZO-1 (Figure S1) but the reduction of vimentin through liver development were found (Figure 1B and 1D; Figure S1; Table 1). Similarly, an increasing of E-cadherin while decreasing of N-cadherin levels were observed (Figure 1C). N- and E-cadherin co-expressing cells were not found in adult liver (Figure 1C), demonstrating that not all the hepatocytes but cells around the central vein express N-cadherin, in consistent with the previous study [25]. Vimentin-positive cells in adult liver are only located near the portal vein, showing that blood vessel comprises these cells (Figure 1B; Figure S1). Statistically, in ED11.5 and ED13.5 liver, most non-hematopoietic cells are both epithelial and mesenchymal (CK8/18^+^vimentin^+^) (Figure 1B), at a rate of 68.3±16.3% and 37.0±29.8%, respectively (Figure 1D; Table 1), in contrast to those of 25.8±7.1% in ED17.5 and 6.0±3.2% in adult liver (Figure 1B and 1C; Table 1). These versatile results provide evidence that mesenchymal and epithelial co-expressing cells decreased with the development stages of mouse liver.

**Table 1:** Dynamic expression of hepatic cell types in liver development.

		E11.5	E13.5	E17.5	Adult
Vim+		69.1±19.1^**^	40.9±7.9^*^	24.9±15.0	6.0±5.5
CK8/18+		73.8±24.4	89.7±8.9	72.5±19.0	100
AFP+		71.4±6.1	69.9±1.1	18.7±2.1^**^	0^*^
BrdU+		88.1±4.9^**^	42.9±3.9^**^	37.1±2.9	0.4±0.4^**^
Vim+	CK8/18+	68.3±21.0^ab^	39.1±18.9	25.8±7.1^a^	6.0±3.2^b^
+	-	0.8±1.4	1.9±2.6	0	0
-	+	5.5±7.1^*^	52.6±24.5	46.7±12.8	94.0±3.2^*^
Vim+	AFP+	69.1±6.4^bc^	39.6±5.5^de^	17.5±1.8^bd^	0^ce^
+	-	0	2.7±2.7	14.8±1.7	6.0±3.2
-	+	6.9±4.3	30.3±8.6^bc^	1.2±0.7^b^	0^c^
Vim+	BrdU+	69.1±6.3^**^	40.9±3.2^a^	24.9±4.7	6.0±3.2^a^
+	-	0	2.1±2.1	0.3±0.3	94.0±2.3^**^
-	+	20.5±4.8	2.0±2.0	18.9±8.0	0

Four-group comparisons are labeled with ^*^(p<0.05) and ^**^(p<0.01).

Two-group comparisons are labeled with the same alphabet a (p<0.05) and b, c, d or e (p<0.01).

Percentage of vimentin, CK8/18, AFP and/or BrdU expressing cells in non-hematopoietic liver cells from mice at indicated development stages. Statistical analyses were done in different liver development.

**Figure 2:**
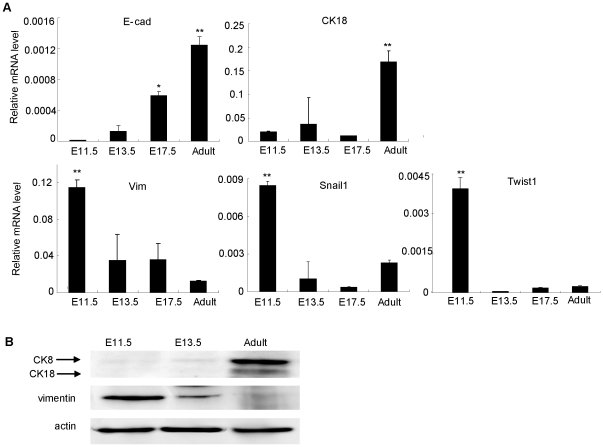
*In vivo* quantitative assay for mesenchymal−epithelial transition in liver developing of mouse. (A) Relative mRNA expressions showed the increasing of Cdh1 (E-cadherin), CK18, and decreasing of vimentin, Snail1 and Twist1 at different developmental stages. RNA was extracted from non-hematopoietic liver cells of C57BL/6J mice. Error bars represented standard errors in three independent experiments. *P<0.05, **P<0.01. (B) Western blot for CK8/18 increasing and vimentin decreasing in non-hematopoietic liver cells of E11.5, 13.5 and adult mice. Actin was used as an internal control. E-cad: E-cadherin.

After removing the hematopoietic cells, adult mouse liver showed a significant increasing of mRNAs encoding epithelial genes CK18 and E-cadherin by 107.8-fold and 8.5-fold, respectively, but remarkable reducing levels of vimentin, Snail1 and Twist1 gene by 9.4-fold, 3.7-fold, and 16.5-fold, respectively, to ED11.5 liver (Figure 2A). Similarly, CK8/18 protein was upregulated from ED11.5 to adult liver with a downregulation of vimentin protein (Figure 2B). These results indicate that MET naturally occurs over the course of mouse liver development.

### Hepatic stem cells isolated from fetal liver co-express both epithelial and mesenchymal markers

**Figure 3:**
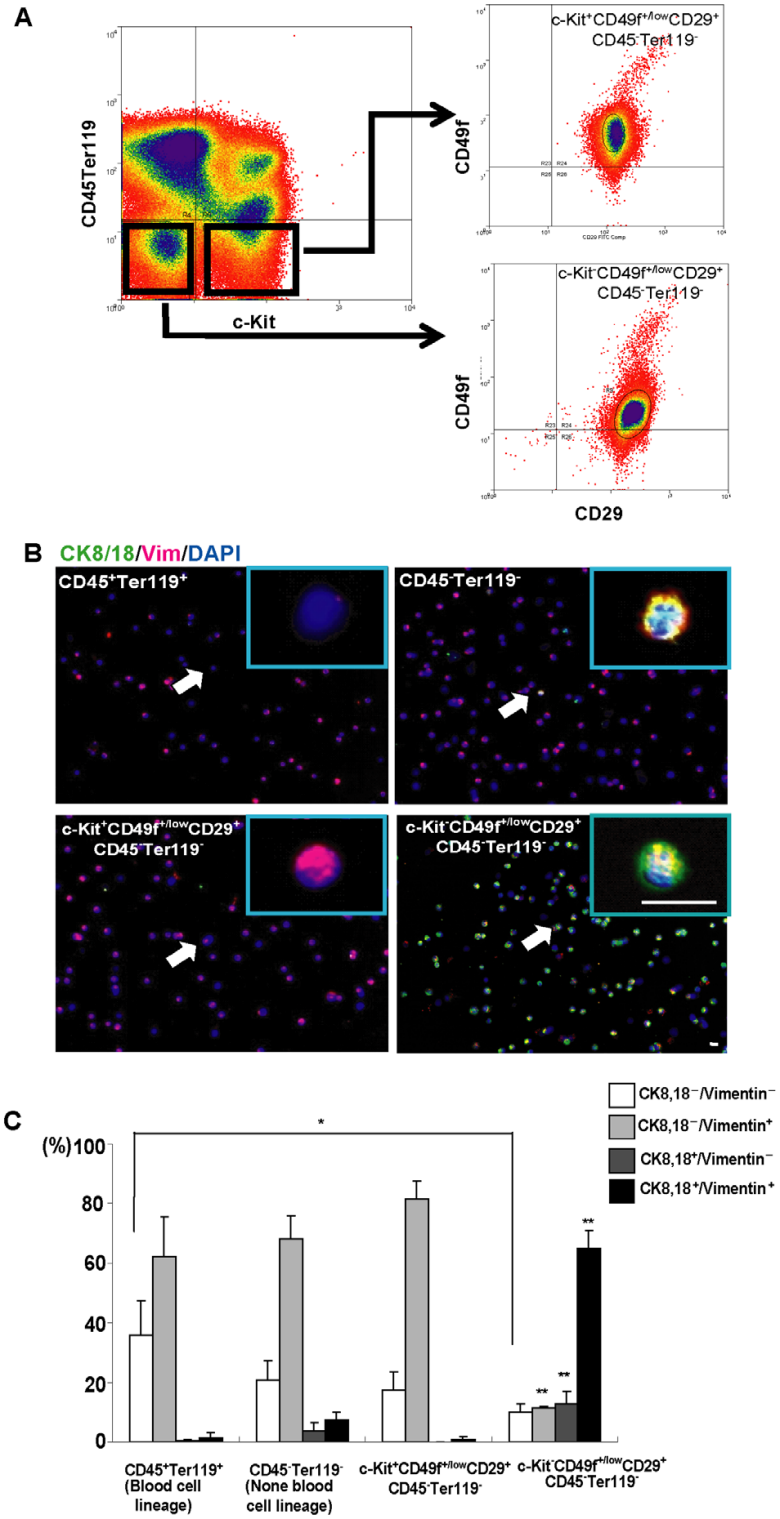
Isolation and characterization of hepatic stem cells of mouse. (A) Hepatic stem cell sorting with flow cytometry. E13.5 mouse liver cells were sorted with gates set for c-kit^−^CD29^+^CD49f^+^CD45^−^TER119^−^ in order to isolate hepatic stem cells as mentioned in Materials and Methods. (B) Immunofluorescence for CK8/18 and vimentin expression in different isolated cell fractions with flow cytometry: CD45^+^Ter119^+^cells (hematopoietic cells), CD45^−^Ter119^−^cells (non-hematopoietic cells), c-Kit^+^CD49f^+/low^CD29^+^CD45^−^Ter119^−^ cells and c-Kit^−^CD49f^+/low^CD29^+^CD45^−^Ter119^−^ cells (hepatic stem cells). Samples were collected by cytospin. (C) Quantifications of CK8/18- and vimentin-expressing cell distributions in the different isolated cell fractions described in (B). Note that the CK8/18^+^vimentin^+^ co-expression cells were main population in stem cell fraction. Error bars represent standard errors in three independent experiments. Arrows showed the cell in magnified pictures. Scheffe's F test. *P<0.05, **P<0.01. Scale bars = 20 µm.

After confirming that MET occurs in the developing mouse liver, we attempted to uncover the relationship between MET and hepatic stem cells, which are related to liver development. In this work we sorted mouse hepatic stem cells from ED13.5 mouse liver; these stem cells can form colonies *in vitro* and reconstruct liver *in vivo*, consistent with previous reports [24]. Using this method, we separated mouse embryonic liver cells into 4 fractions: CD45^+^TER119^+^ cells (hematopoietic cells), CD45^−^TER119^−^ cells (non- hematopoietic cells), c-Kit^+^CD49f^+/low^CD29^+^CD45^−^TER119^−^ cells, and c-Kit^−^CD49f^+/low^CD29^+^CD45^−^TER119^−^ cells, which we considered to be the fetal liver stem cell fraction (Figure 3A). To figure out which fraction of cells expressed the epithelial and/or mesenchymal characteristics, we stained these cells for CK8/18 and vimentin using the Cytospin. As anticipated, of the 4 sorted cell fractions, the stem cells had the highest percentage (65.0%) of CK8/18 and vimentin double positive; this level was 43.3-fold higher than for hematopoietic cells (1.5%), 8.8-fold higher than non-hematopoietic cells (7.4%), and 59.1-fold higher than c-Kit^+^CD49f^+/low^CD29^+^CD45^−^TER119^−^ cells (1.1%) (Figure 3B and 3C). Thus, the CK8/18 and vimentin double-positive population are mainly found in the stem cell fraction, which is significantly different from other cell fractions.

Among the c-Kit^−^CD49f^+/low^CD29^+^CD45^−^TER119^−^ cells, the CK8/18-positive cells are mainly found at a high frequency as 78.0%, whereas the frequencies were very low in other fractions (1.9% in hematopoietic cells; 11.1% in non-hematopoietic cells; 1.1% in c-Kit^+^CD49f^+/low^CD29^+^CD45^−^TER119^−^ cells). Besides CK8/18, other epithelia markers such as E-cadherin and ZO-1, the hepatic lineage marker albumin, the bile duct marker CK7, and mesenchymal marker N-cadherin were all detected in the isolated stem cells (Figure S2), showing co-expression of epithelial and mesenchymal molecules in stem cells. These data demonstrate that sorted c-Kit^−^CD49f^+/low^CD29^+^CD45^−^TER119^−^ hepatic stem cells are mainly CK8/18 positive and of mesenchymal character, and thus are distinct from other cells in fetal liver.

### Mesenchymal-epithelial transition occurs in stem cell-derived colonies

**Figure 4:**
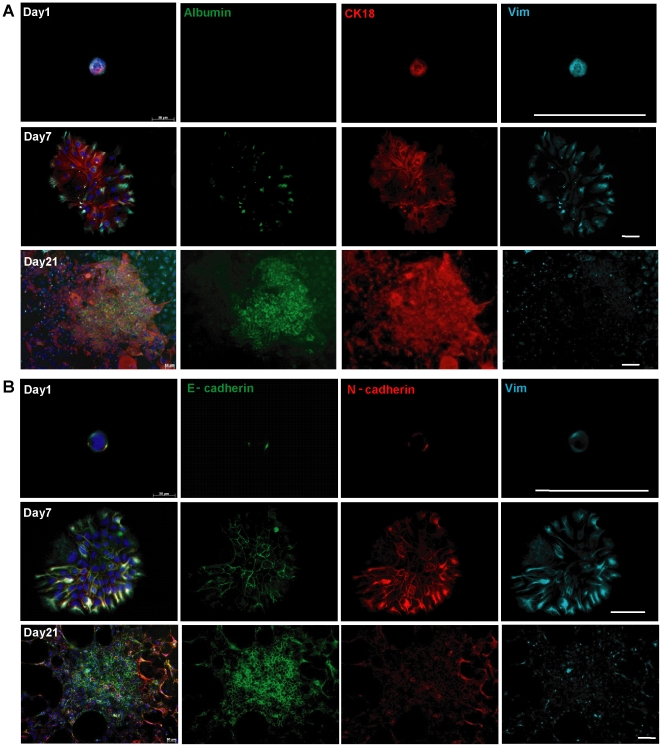
Mesenchymal−epithelial transition occurred in stem cell-derived colonies during culture. Immunofluorescence for stem cells and stem cell-derived colonies at indicated culture days. (A) CK8/18 and vimentin transition accompanied with hepatic differentiation of stem cells into albumin positive cells; (B) E-cadherin and N-cadherin, vimentin transition accompanied with hepatic differentiation of stem cells. Scale bars = 100 µm.

To determine whether the mesenchymal and epithelial hepatic stem cells could experience MET events during stem cell differentiation, we cultured isolated stem cells for clonal colony assay and gene expression. Colonies on culture day 21 showed more expanded staining for epithelial markers albumin, CK8/18, CK7, ZO-1 and E-cadherin but less vimentin and N-cadherin than cells on day 0 (Figure 4 and Figure S3). Moreover, co-expression of CK8/18 and CK7 was detected in stem cell-derived colonies (Figure S3B), indicating biliary differentiation of CK18-expressing cells. Even on day 21, there are still cells double positive for albumin and CK7 (Figure S3D), meaning that they have hepatocyte and bile duct dual potential.

**Figure 5:**
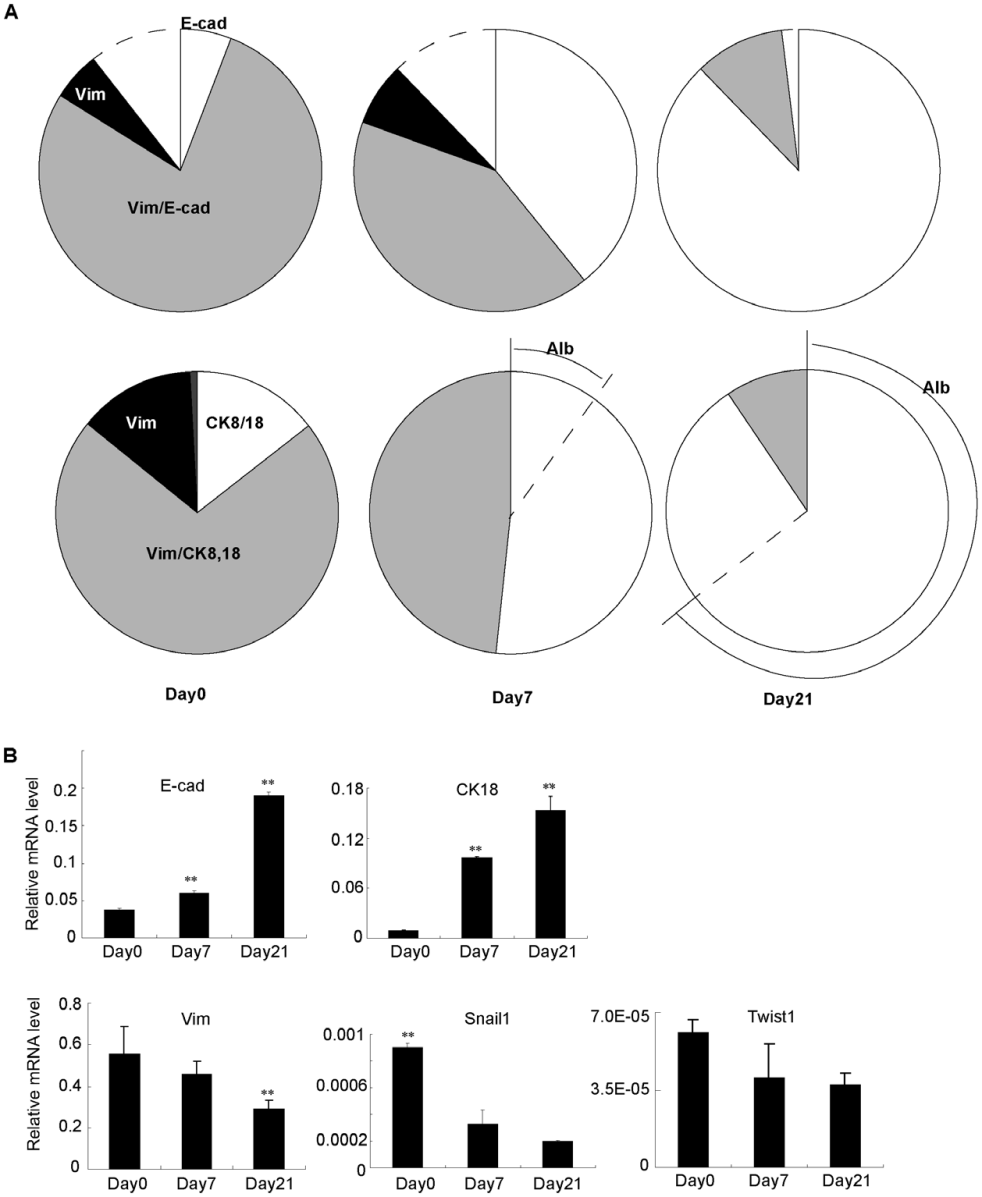
*In vitro* quantitative assay for mesenchymal−epithelial transition of stem cell-derived colonies. (A) Quantification of immunofluorescence in cultured stem cells on day 0 and stem cell derived colonies on days 7 and 21. Upper panel, E-cadherin and vimentin expressing cell assay (dashed regions indicate double-negative cells); lower panel, CK8/18, vimentin and albumin assay. Graph showed quantification of three independent experiments. (B) Relative mRNA expressions in cultured stem cells and stem cell derived colonies. It showed up-regulation of epithelial genes, Cdh1 (E-cadherin) and CK18, and down-regulation of mesenchymal genes, vimentin, Snail1 and Twist1 during stem cell differentiation according to the passing by of culture time. Error bars represent standard errors in three independent experiments. **P<0.01. Alb: albumin. E-cad: E-cadherin.

To further characterize MET in stem cell differentiation, we performed the quantitative assay for the dynamic changes of E-cadherin and vimentin, CK8/18 and vimentin expressions in stem cell-derived colonies in culture (Figure 5). Notably, the frequency of mesenchymal (vimentin^+^) cells decreased from 84.0% on day 0 of culture to 48.3% on day 7 and 9.2% on day 21 (Figure 5A). In contrast, epithelial (E-cadherin^+^) cell frequency increased from day 0 (83.8%) to day 21 (98.1%); and CK8/18 positive cell proportion increased significantly, from 85.0% on day 0 to 100% on both day 7 and day 21, respectively (Figure 5A). Of these cells, the relative quantity of vimentin and E-cadherin co-expressing cells decreased from day 0 (77.9%) to day 7 (41.3%) and day 21 (10.3%); similarly, vimentin and CK8/18 double-positive cells decreased profoundly from day 0 (71.3%) to day 7 (48.3%) and day 21 (9.2%) (Figure 5A) (*P*<0.05). Furthermore, the albumin^+^ hepatocytes were all CK8/18 positive; these cells were first observed on day 7 (9.0%), and increased by day 21 (65.0%) (Figure 5A). Additionally, the differentiated hepatic cells (CK18^+^ and albumin^+^) in each colony are surrounded by the mesenchymal-like cells (CK18^+^ and Vimentin^+^) in the periphery of the colony (Figure 5), suggesting that centrally located cells undergo MET and become differentiated earlier than those in peripheral sites.

Relative gene expression measurements in stem cell-derived colonies confirmed and extended the results of immunocytochemistry, demonstrating upregulation of E-cadherin and CK18, but downregulation of vimentin, Snail1 and Twist1 (Figure 5B). Both CK18 and E-cadherin (Cdh1) gene expression increased significantly from day 0 to day 7 (10.3-fold for CK18 and 1.7-fold for E-cadherin) and from day 7 to day 21 (1.6-fold for CK18 and 3.2-fold for E-cadherin) while vimentin and Snail1 except Twist1 demonstrated downward trend in expression between day 0 and day 21 (1.9-fold for vimentin and 4.5-fold for Snail1) (Figure 5B). From the results of the colony assay and gene expression measurements, we conclude that mesenchymal hepatic stem cells experience MET during hepatocyte and biliary cell differentiation.

### Vimentin positive mesenchymal fetal liver cells are highly proliferative *in vivo*

**Figure 6:**
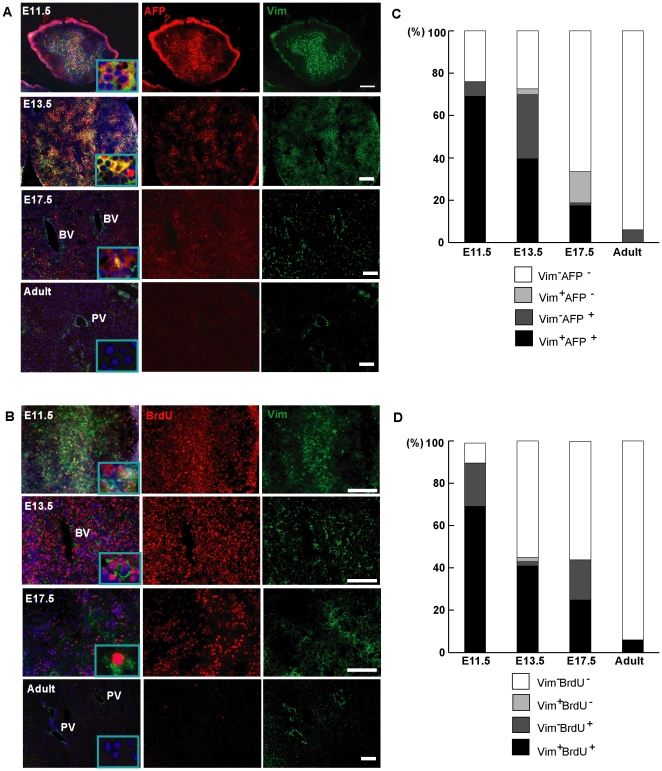
Vimentin-positive mesenchymal fetal liver cells are highly proliferative *in vivo*. (A) Representative images of dual immunofluorescence of AFP and vimentin; (B) BrdU and vimentin in mice livers at different developmental stages. AFP and BrdU expressions decreased accompanied with vimentin reduction. (C) and (D) represented the relative quantitative assay of non-hematopoietic cells in (A) and (B) respectively. These results showed that AFP positive liver cells also expressed vimentin, and the vimentin^+^ cells are highly proliferative (BrdU^+^). Scale bars = 100 µm.

According to the results described above (Figures 1, 2, 3, 4, 5), hepatic stem cells and a large population of fetal liver cells both cell types have the capacity to proliferate and differentiate into mature liver cells, and more importantly, express vimentin. AFP was first described as a hepatic lineage gene in embryonic gastrulation [26]. Therefore, to further investigate the correlation of vimentin and cell proliferative ability, we continued to characterize vimentin-positive fetal liver cells co-expressing AFP and BrdU, after removal of hematopoietic cells with CD45 and TER119 antibodies. As shown in Figure 6A, frequencies of vimentin and AFP were both very high at ED11.5 comparing with adult mouse liver (Figure 6A and 6C; Table 1). Almost all the vimentin-positive cells are AFP-positive at ED11.5 (100%) and ED13.5 (94.7±9.2%) mouse liver (Figure 6C), yet the percentage of AFP-positive in vimentin-expressing cells decreases dramatically at ED17.5 (15.0±10.2%) and disappears altogether in adult mouse liver (Figure 6C; Table 1). It is worth noticing that almost all the vimentin-positive cells are BrdU-positive in fetal mouse liver (ED11.5: 100%, ED13.5: 95.7±10.5%, ED17.5: 95.7±7.5%) (Figure 6D), as opposed to almost no BrdU-positive cells in adult mouse liver (0.5±0.9%). Thus, during the developing of mouse liver, the number of vimentin-positive cells decreases; meanwhile, the proportion of vimentin-positive cells that are also AFP- or BrdU-positive decreases as well (Table 1; Figure 6C and 6D). Taken together, these measurements provide an *in vivo* indication that in mouse liver of ED11.5 and ED13.5, hepatoblasts have mesenchymal character (vimentin^+^) with proliferative activation, whereas in ED17.5 and adult mouse liver, the number of activated cells decreases dramatically concomitant with vimentin reduction. Vimentin, on one hand, has high co-expression with AFP, suggesting that vimentin is associated with immaturity of the liver; on the other, vimentin is also linked to cell proliferation, in that the occurrence of vimentin is related to the liver tissue activity in the developing process.

## Discussion

### Different stem cells, including hepatic stem cells, have mesenchymal characters

In this study, we confirmed that MET occurs during liver development between ED11.5 and adulthood in the mouse. Furthermore, by using fluorescence-activated cell sorting, we obtained evidence that hepatic stem cells, isolated from fetal liver, spontaneously experience MET during the differentiation into hepatocytes and biliary cells *in vitro*. Importantly, distinct from other cells isolated from fetal liver, hepatic stem cells have both epithelial and mesenchymal characteristics.

Consistent with our results, stem cells derived from different sources express mesenchymal characters. For example, stem cells in epithelial tissue, e.g., mammary stem cells, have been confirmed as mesenchymal-like [3], [27]. This has also been confirmed in mesenchymal tissue, e.g., muscle stem cells and neural stem cells [28]–[30]. Moreover, embryonic stem cells also have a mesenchymal phenotype in the gastrulating embryo [31]. Furthermore, cancer-initiating cells or cancer stem cells in invasive tumors — e.g., intestine cancer stem cells [32], mammary cancer stem cells [3] and prostate cancer stem cells [33] — also have mesenchymal features. The results reported here may indicate that mesenchymal capability is necessary for stem cells derived from various sources to easily proliferate, loosen cell−cell connection, migrate and differentiate.

### Cell activation is associated with vimentin expression

By detecting cells in S phase, BrdU staining can identify proliferative cells. In our AFP/vimentin and BrdU/vimentin co-staining experiments, we reveal that the AFP-expressing liver cells are mesenchymal; furthermore, these cells are in a highly proliferative state. These results suggest that besides as a marker in normal and abnormal mesenchymal cells [2], [34], [35], vimentin has a novel role in connection with AFP^+^ cells and BrdU^+^ cells, indicating these cells are activated for proliferation. Indeed, vimentin appears to be related to the activation of mesenchymal cells: for example, in vimentin-deficient mice, the motility and migration of fibroblasts are impaired [23]. In another report, vimentin-deficient cells exhibited decreased stiffness as well as decreased DNA synthesis [36]. More recently, vimentin has been regarded as mesenchymal cell marker in cancer-related EMT; drugs that target cancer cell growth also induce vimentin degradation in cancer cells [37]. These results support our finding that vimentin is related to cell activation in cancer as well as in normal tissue.

In addition to being associated with migration and proliferation of mesenchymal cells, vimentin is an indicator of cell morphology transformation or cytoskeleton reorganization [38], [39]. In mouse embryonic gastrulation, vimentin increases in fibroblasts that delaminate through the primitive streak to become mesoderm [23], [40], [41], indicating that vimentin plays a role in cell transformation and tissue construction. Moreover, vimentin is closely related to loss of polarity of the plasma membrane in fiber cells [42], cell adhesion and polarization are associated with decreasing vimentin [43]. Furthermore, in metastatic cancer, epithelial cancer cells experience EMT concomitant with upregulation of vimentin as they disassociate from primary tumors, transform into mesenchyme, and migrate. These mesenchymal cells then undergo MET concomitant with downregulation of vimentin at metastasis sites [3], [16], [44]–[46]. Interestingly, it is reported that cytoplasmic vimentin and keratin5/14 share a similar coil structure, though the function of this similarity is not clear [47], suggesting the possibility of a vimentin-to-keratin transition within the same cell, consistent with our observations of MET. The reorganization of vimentin molecular structure in the cytoplasm may explain the plasticity of stem cells and the transformation of mesenchymal cells into epithelial cells.

**Figure 7:**
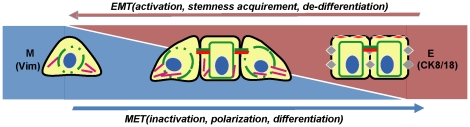
A Schematic model for MET. MET is involved in stem cell inactivation, cell polarization and differentiation. This process is associated with a reduction of vimentin and accumulation of CK8/18 in stem cells. Furthermore, the variation of vimentin in stem cells is an indicator of cell proliferative activity. Conversely, it is suggested that EMT causes cells into active and de-differentiated state and acquire stem cell-like characteristics. M: mesenchymal state; E: epithelial state; Vim: vimentin.

Although the link between stem cells and vimentin-positive cells in fetal liver has not been completely elucidated, we suggest that vimentin is a critical indicator for activated cells experiencing MET. Accordingly, because of the elastic state of stem cells, our results provide a hint that MET in stem cells indicates rearrangement of cytoskeleton as well. During the process of inactivating mesenchymal cells or the developing fetal liver, the number of vimentin-positive cells decreased (Figures 6 and 7).

### Mesenchymal epithelial transition generates cell inactivation

In this study of MET, the adult liver expressed very low rate of vimentin, AFP and BrdU positive, suggesting that adult liver cells are in quiescent state. Based on the preceding statements, it can be inferred that MET drives stem cells into a quiescent state, whereas EMT is associated with reactivation or reprogramming of epithelial cells [3], [45] (Figure 7). MET is also involved in other cell inactivation, for example, in wound healing, activated fibroblasts lose cell polarity, migrate into the wound site and differentiate into keratinocytes [23], a process driven by MET. During metastasis tumor invasion, epithelial tumor cells become mesenchymal-like and disassociate from the primary tumor, indicating that epithelial cell activation and proliferation [17] are driven by EMT. Subsequently, those de-differentiated mesenchymal cells colonize and differentiate into secondary carcinomas [17]. Comparing with invasive sites, the central area of many metastases showed reduced expression of nuclear β-catenin [48], indicating the inactivated state of the MET driven cells. Take together, it is suggested that in stem cell differentiation, wound healing and malignant tumor [32], MET process is related with cells changing from mesenchymal, de-polarized, proliferative and migrating state, to epithelial, polarized, differentiated, adhesive and inactivation state.

### Mesenchymal epithelial transition in hepatic stem cell reflects a natural process in vitro

There is still some controversy about the relationship between the tissue microenvironments and EMT. One group claimed that cytokines such as EGF and HGF in cell culture can induce epithelial cells' transformation into mesenchymal-like cells [49]. In addition, other findings in pancreatic cells suggest that EMT is not involved in the origin of pancreatic mesenchymal stem cells (MSCs) [50]. In their report [50], EMT depended on the culture condition, and some of the vimentin single-positive cells are directly derived from the mesenchymal cells which are not experiencing EMT. Whether MET is influenced by these microenvironments is still unknown. In contrast, on one hand, we confirmed that MET occurs during mouse liver development both *in vivo* and *in vitro* (Figures 1, 2, 3, 4, 5). On the other hand, in stem cell colonies, the frequency of vimentin-positive cells decreased over the course of time in culture, and vimentin-positive but CK8/18-negative cells disappeared after day 7 (Figures. 4 and 5A). These dynamic transitions indicate that the vimentin-positive mesenchymal cells differentiated into epithelial cells. Additionally, we found that differentiated hepatocytes (albumin-positive) are derived from CK8/18- and vimentin-double-positive cells (Figures 4 and 5A), demonstrating that MET occurs naturally in stem cell differentiation.

### Conclusion

In summary, from the view of MET in this study, our results confirm the concept that MET is a normal process associated with stem cell differentiation and liver development. This may offer the promise of specifying the hepatic stem cells in liver regeneration and chronic liver injury, in which MET/EMT are involved. More importantly, to our knowledge, direct evidence shows that in addition to serving as a marker for mesenchymal cells, vimentin can also reveal cell activation and proliferation state. These results provide further insights that will help to elucidate the effects of vimentin in hepatic stem cell differentiation and progression of liver pathology.

## Materials and Methods

### Animals and cell preparation

C57BL/6J mice at embryonic days (ED) 11.5, 13.5, 17.5 and 8 weeks (Adult) after birth were purchased from Japan SLC (Tokyo, Japan). All animal experimentation was conducted in accordance with the Guidelines for Proper Conduct of Animal Experiments (Science Council of Japan), and all protocols were approved by institutional review board of Animal Research Center, Yokohama City University School of Medicine (09-48). For the *in vitro* fluorescent activated cell sorting (FACS)® assays, single cell suspensions of liver cells were prepared from fetal mice. Embryonic liver was treated with 0.1% trypsin−1 mM ethylene glycol tetraacetic acid (EGTA) in order to dissociate cells, as described [51].

### Cell sorting and culture

ED13.5 fetal mouse liver cells were incubated at 4°C for 30 minutes with biotinylated anti-CD45 (PharMingen, San Jose, CA) and TER119 mAb (PharMingen) phycoerythrin-conjugated anti-CD49f mAb (PharMingen), fluorescein isothiocyanate- conjugated anti-CD29 mAb (PharMingen), allophycocyanin-conjugated anti-c-Kit mAb (PharMingen). After 3 washings with staining medium (3% FCS in PBS), cells were stained with streptavidin-labeled APC-Cy7 (PharMingen) at 4°C for 20 minutes. Finally, cells were washed 3 times and resuspended in staining medium containing propidium iodide (1 µg/mL) (Sigma, St Louis, MO). Labeled cells were analyzed and separated with MoFlo (DakoCytomation, Glostrup, Denmark) and Summit version 4.0 software (DakoCytomation). After removal of hematopoietic cells by CD45 and TER119 antibodies, four cell fractions are compared, CD45^+^TER119^+^, CD45^−^TER119^−^, c−kit^+^CD49f^+^CD29^+^CD45^−^TER119^−^, and c−kit^−^CD49f^+^CD29^+^CD45^−^TER119^−^ subpopulation which are reported as hepatic stem cell fraction [24]. Cells were cultured on type IV collagen coated 6-well plates (Becton Dickinson, San Jose, CA) at a density of 500cells/cm^2^ in our standard culture medium [51]. Human recombinant hepatocyte growth factor (HGF) (50 ng/mL) (Sigma, St Louis, MO) and epidermal growth factor (EGF) (20 ng/mL) (Sigma) were added 24 hours after culture initiation. Cells on culture days 0, 1, 7 and 21 are collected for relative mRNA expression and immunofluorescence analyses.

### Histology and immunofluorescence

Tissues were fixed with 10% neutral formalin and embedded in paraffin. Histological 3-µm-thick serial cross sections were cut at 50-µm intervals and stained with hematoxylin and eosin. Cytospin samples were sorted on MAS coated glass slides (Matsunami glass, Japan), followed by centrifugation at 2000rpm, 4°C for 5 min. For *in vivo* assays, C57BL/6J mouse livers from different developmental stages were embedded in Tissue-Tek® OCT compound (Sakura Finetek, Japan). For immunofluorescence assays, Cytospin, cultured cells and cryostat liver tissue sections were fixed with 2% Paraformaldehyde (PFA) at 4°C for 20 minutes, and washed in PBS including 0.05% Tween 20 (Wako). Nonspecific binding was blocked with 10% nonimmune serum of a species from which the secondary antibody had been obtained. Samples were incubated with primary antibodies and second antibodies as described in Table S1. After final washing, cells were nuclei were stained blue with DAPI and viewed with a ZEISS Axio Imager.M1 microscope.

### RNA isolation and Real-time PCR

Liver cells were treated as described above. Non-hematopoietic cells were collected after the removal of CD45^+^TER119^+^ labeled hematopoietic cells by flow cytometry. Total RNA was isolated with ISOGEN (Nippon gene, Tokyo, Japan), treated with DNaseI (Invitrogen, Carlsbad, CA) to get rid of genome DNA and complementary DNA (cDNA) was synthesized using the SuperScriptIII Reverse Transcriptase (Invitrogen), as described [24]. Quantitative reverse transcript PCR analysis was performed using the ABI PRISM7700 real-time PCR system (Applied Biosystems, Foster city, CA). Expression levels were normalized to that of glyceradehyde-3-phosphate dehydrogenase (GAPDH). TaqMan PCR probes (Applied Biosystems) are listed in Table S2.

### Western blotting

Non-hematopoietic liver cells (CD45^−^TER119^−^) were isolated with IMag Streptavidin Particles Plus (BD Biosciences, San Jose, CA) under manufacture's instructions. Cells were lysed in the presence of 50 mM Tris, pH 7.5, 150 mM NaCl, 0.1% SDS, 0.5% Deoxycholic acid sodium salt and 1% NP-40 on ice. Thirty micrograms of total protein from each sample was fractionated on a 10% Acrylamide Gel and transferred to PVDF membranes. Blots were subjected to anti-vimentin (Sigma, St Louis, MO), anti-CK8/18 (PROGEN, Germany) and anti-β-actin (Cell Signaling, Beverly, MA) antibodies. Proteins were visualized using the ECL detection reagent from Amersham (GE Healthcare, UK).

### DNA synthesis assay

BrdU (50 µg/kg body weight) (Sigma) dissolved in PBS with 7 mM NaOH was injected intraperitoneally into mice 6 h before sacrifice. After being fixed with 2% PFA and washed with 0.05% Tween 20 in PBS, liver frozen sections were treated with 2N HCl and neutralized in 0.1 M sodium tetraborate (pH 8.5). The sections were then stained with anti-BrdU antibody (BD Pharmingen, San Jose, CA) as described [52] using Alexa Fluor®488 goat anti-mouse IgG_1_ (Invitrogen, Carlsbad, CA) as a secondary antibody for visualization.

### Statistical analysis

All data are presented as mean±SD. Scheffe's F test and Bonferroni/Dunn correction were used to compare multiple groups of data. *P*<0.05 and *P*<0.01 were considered as statistically significant and great significant respectively.

## Supporting Information

Figure S1 **Mesenchymal−epithelial transition occurs in mouse liver development.** Representative images of dual immunofluorescence of epithelial (ZO-1) and mesenchymal (vimentin) cells in C57BL/6J mice livers at different developmental stages. Scale bars = 100 µm.
(TIF)

Figure S2 **Hepatic stem cells are both epithelial- and mesenchymal-like.** (A) Immunofluorescence of hepatocyte marker albumin, biliary cell marker CK7 and vimentin; (B) CK8/18, CK7 and vimentin; (C) E-cadherin, N-cadherin and vimentin; (D) E-cadherin, ZO-1 and vimentin expression in isolated c-Kit^−^CD49f^+/low^CD29^+^CD45^−^Ter119^−^ hepatic stem cells with flow cytometry. Scale bars = 50 µm.
(TIF)

Figure S3 **Mesenchymal−epithelial transition occurs in stem cell-derived colonies during culture.** (A) During culture day 1 to day 21, stem cells and stem cell-derived colonies were immunostained with albumin, CK7 and vimentin; (B) CK8/18, CK7 and vimentin; or (C) E-cadherin, ZO-1 and vimentin. (D) Partially differentiated stem cell colonies were indicated. Arrowhead, albumin and CK7 co-expressing cells. Scale bars = 100 µm.
(TIF)

Table S1 **List of Antibodies.** 1^st^ and 2^nd^ antibodies used in the immunofluorescence experiments are listed.
(DOC)

Table S2 **List of TaqMan probes.** TaqMan probes used for detection of the relative gene expressions are listed.
(DOC)
