# Supplementary figures and images for: Evidence for Mesenchymal−Epithelial Transition Associated with Mouse Hepatic Stem Cell Differentiation
Source: PLoS One. 2011 Feb 11;6(2):e17092. doi: 10.1371/journal.pone.0017092 (PMC3037942; doi:10.1371/journal.pone.0017092)

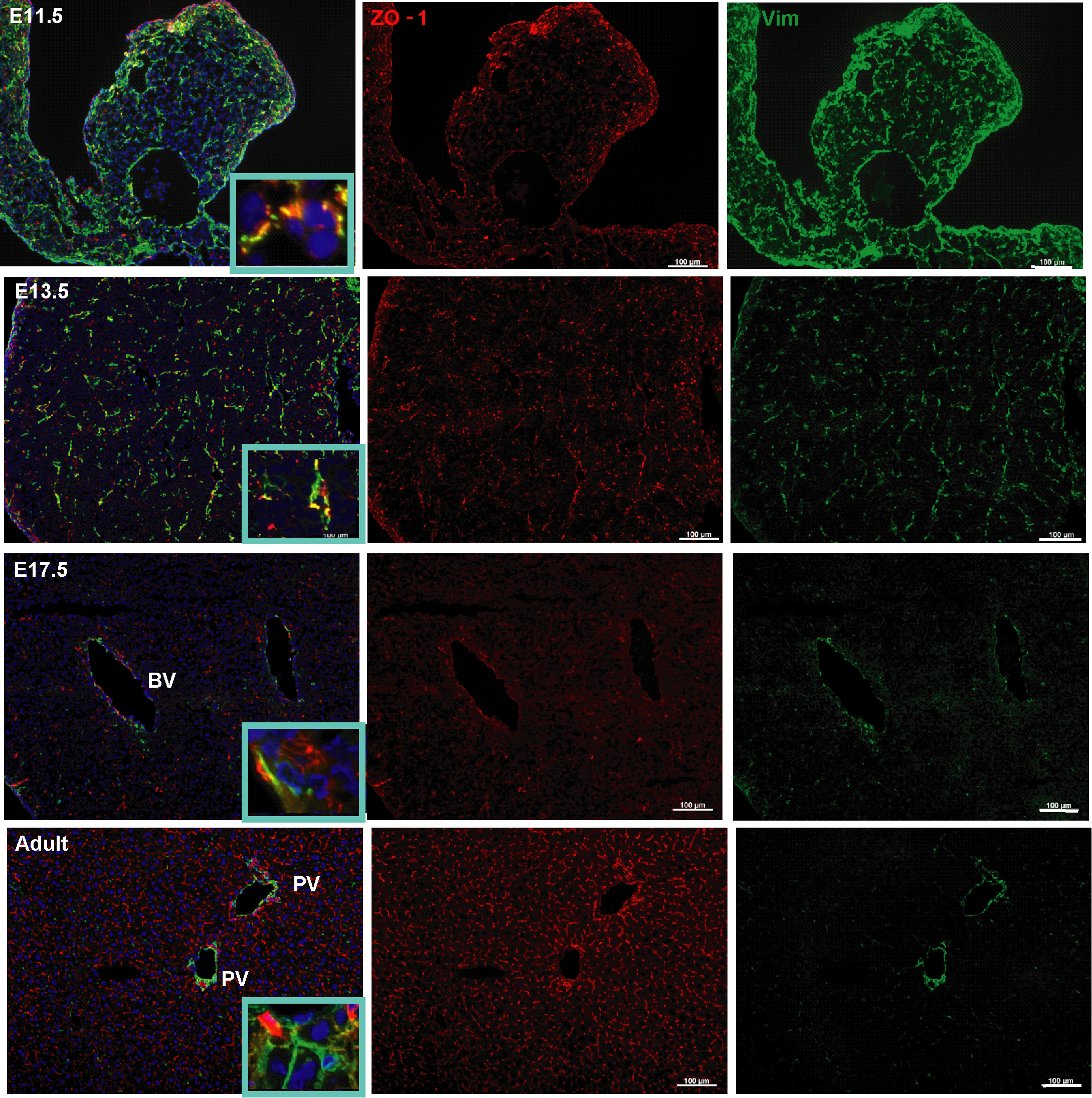

Supplement: Figure S1 — Mesenchymal−epithelial transition occurs in mouse liver development. Representative images of dual immunofluorescence of epithelial (ZO-1) and mesenchymal (vimentin) cells in C57BL/6J mice livers at different developmental stages. Scale bars = 100 µm. (TIF) [file pone.0017092.s001.tif]

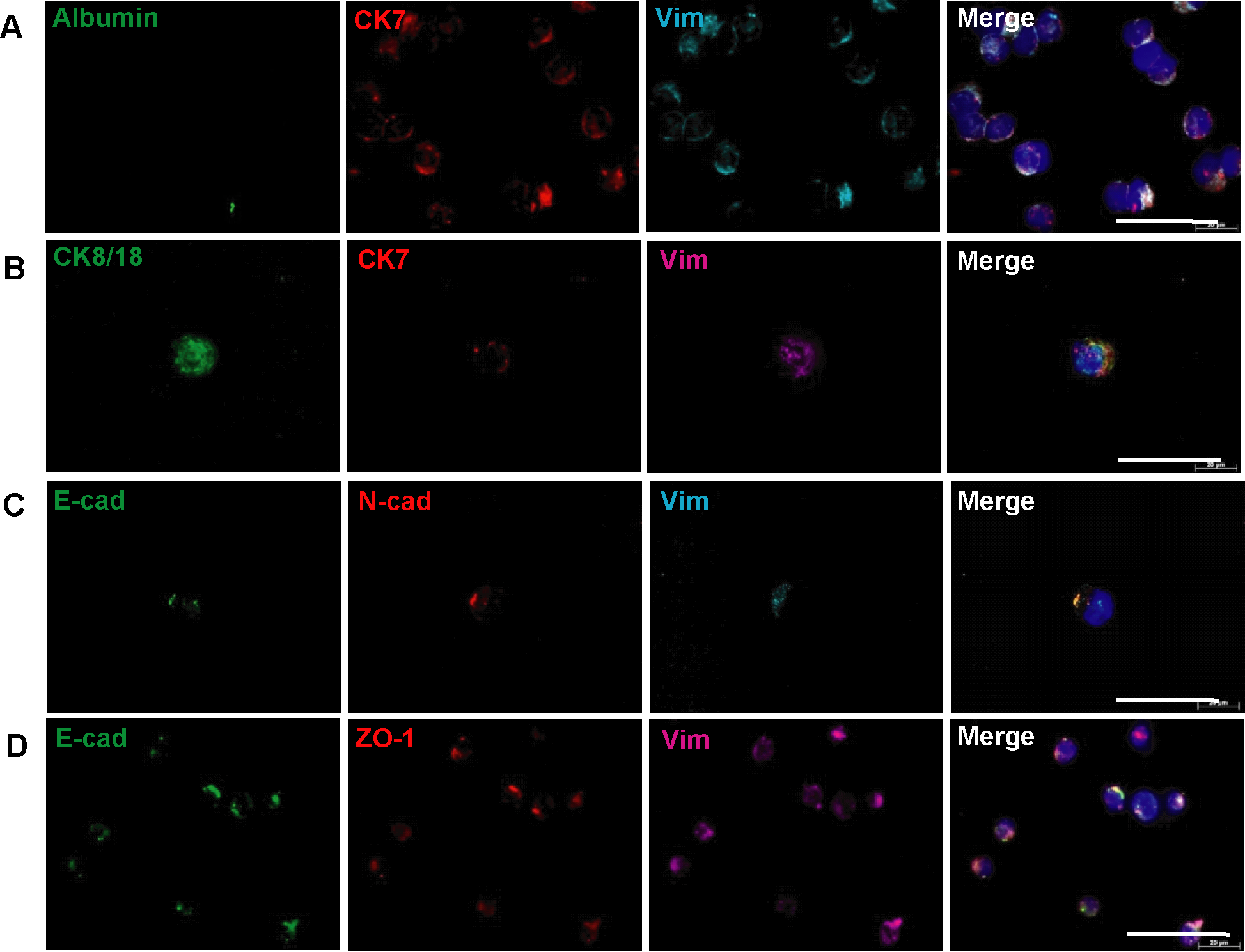

Supplement: Figure S2 — Hepatic stem cells are both epithelial- and mesenchymal-like. (A) Immunofluorescence of hepatocyte marker albumin, biliary cell marker CK7 and vimentin; (B) CK8/18, CK7 and vimentin; (C) E-cadherin, N-cadherin and vimentin; (D) E-cadherin, ZO-1 and vimentin expression in isolated c-Kit−CD49f+/lowCD29+CD45−Ter119− hepatic stem cells with flow cytometry. Scale bars = 50 µm. (TIF) [file pone.0017092.s002.tif]

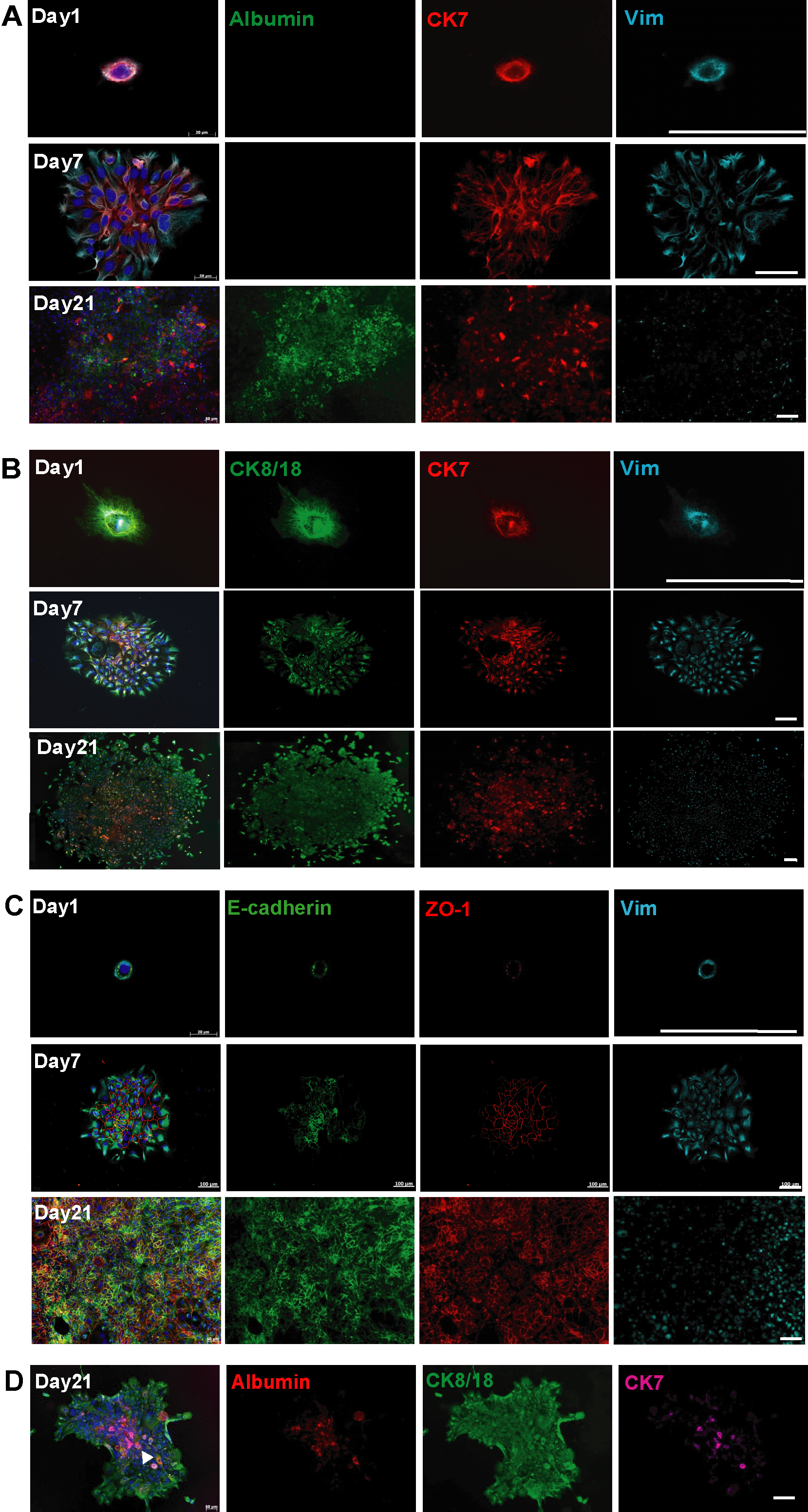

Supplement: Figure S3 — Mesenchymal−epithelial transition occurs in stem cell-derived colonies during culture. (A) During culture day 1 to day 21, stem cells and stem cell-derived colonies were immunostained with albumin, CK7 and vimentin; (B) CK8/18, CK7 and vimentin; or (C) E-cadherin, ZO-1 and vimentin. (D) Partially differentiated stem cell colonies were indicated. Arrowhead, albumin and CK7 co-expressing cells. Scale bars = 100 µm. (TIF) [file pone.0017092.s003.tif]
