# Supplementary material for: Evidence for Mesenchymal−Epithelial Transition Associated with Mouse Hepatic Stem Cell Differentiation
Source: PLoS One. 2011 Feb 11;6(2):e17092. doi: 10.1371/journal.pone.0017092 (PMC3037942; doi:10.1371/journal.pone.0017092)
Supplement: Table S1 — List of Antibodies. 1st and 2nd antibodies used in the immunofluorescence experiments are listed. (DOC) [file pone.0017092.s004.doc]

**Table S1**

Antibodies

| **1^st^Antibody** | **Manufacturer** |
| --- | --- |
| Guinea pig anti-CK8/18 | Progen, Germany |
| mouse anti-cytokeratin7 (CK7) | Dako, UK |
| mouse anti-vimentin | Sigma-Aldrich, St. Louis, MO |
| rabbit anti-albumin (Alb) | Biosciences, UK |
| mouse anti- Zona occludens-1 (ZO-1) | Zymed Laboratories, Carlsbad, CA |
| mouse anti-E-cadherin | BD Transduction Laboratories, San Jose, CA |
| mouse anti-N-cadherin | BD Bioscience |
| rabbit anti-α-fetoprotein (AFP) | MP Biomedicals, Cleveland, OH |
| Biotin rat anti-mouse CD45 | BD Pharmingen, San Jose, CA |
| Biotin rat anti-mouse TER119 | BD Pharmingen |

| **2^nd^ Antibody** |  |
| --- | --- |
| Alexa Fluor®488 goat anti-guinea pig IgG | Invitrogen, Carlsbad, CA |
| Alexa Fluor®488 goat anti-rabbit IgG | Invitrogen |
| Alexa Fluor®488 goat anti-mouse IgG_2a_ | Invitrogen |
| Alexa Fluor®555 goat anti-mouse IgG_1_ | Invitrogen |
| Alexa Fluor®647 goat anti-mouse IgG_1_ | Invitrogen |
| Alexa Fluor®647 goat anti-mouse IgM | Invitrogen |
| Strepavidin, Alexa Fluor®647 | Invitrogen |
| Cy-3 goat anti-rabbit IgG(H+L) | Jackson ImmunoResearch, West Grove, PA |
