# Supplementary material for: Evidence for Mesenchymal−Epithelial Transition Associated with Mouse Hepatic Stem Cell Differentiation
Source: PLoS One. 2011 Feb 11;6(2):e17092. doi: 10.1371/journal.pone.0017092 (PMC3037942; doi:10.1371/journal.pone.0017092)
Supplement: Table S2 — List of TaqMan probes. TaqMan probes used for detection of the relative gene expressions are listed. (DOC) [file pone.0017092.s005.doc]

**Table S2**

**TaqM**an probes

| **Gene** | ID number |
| --- | --- |
| E-cadherin | Mm00486906_m1 |
| Cytokeratin 18 | Mm01601706_g1 |
| Vimentin | Mm01333430_m1 |
| N-cadherin | Mm00483213_m1 |
| Snail | Mm00441533_g1 |
| Twist1 | Mm00442036_m1 |
| GAPDH | Mm99999915_g1 |
